# Can Polyphenols in Eye Drops Be Useful for Trabecular Protection from Oxidative Damage?

**DOI:** 10.3390/jcm9113584

**Published:** 2020-11-06

**Authors:** Sergio Claudio Saccà, Alberto Izzotti, Stefania Vernazza, Sara Tirendi, Sonia Scarfì, Stefano Gandolfi, Anna Maria Bassi

**Affiliations:** 1 Ophthalmology Unit, IRCCS, San Martino General Hospital, 16132 Genoa, Italy; sergio.sacca@hsanmartino.it; 2 Department of Experimental Medicine (DIMES), University of Genoa, 16132 Genoa, Italy; izzotti@unige.it (A.I.); tirendisara@gmail.com (S.T.); Anna.Maria.Bassi@unige.it (A.M.B.); 3 IRCCS-Fondazione Bietti via Livenza 3, 00198 Rome, Italy; 4 Inter-University Center for the Promotion of the 3Rs Principles in Teaching & Research (Centro 3R), Italy; soniascarfi@unige.it; 5 Department of Earth, Environmental and Life Sciences (DISTAV), University of Genoa, 16132 Genoa, Italy; 6 Ophthalmology Unit, Department of Biological, Biotechnological and Translational Sciences, University of Parma, 43121 Parma, Italy; stefano.gandolfi@unipr.it

**Keywords:** iTRAB^®^, 3D-advanced culture model, dynamic culture conditions, trabecular meshwork, oxidative stress, glaucoma, polyphenols, milli-fluidic technology

## Abstract

Polyphenols, with anti-oxidant properties, counteract oxidative stress effects. Increasing evidence has found oxidative stressto be the main risk factor for trabecular meshwork (TM) damage, leading to high-tension glaucoma. Topical anti-oxidants could represent a new target for glaucoma treatment. Our aim is to investigate the protective mechanisms on a human TM culture of a patented polyphenol and fatty acid (iTRAB^®^)formulation in response to oxidative stress using an advanced invitromodel consisting of 3D-human TM cells, embedded in a natural hydrogel, and a milli-scaled multi-organ device model for constantdynamic conditions. The 3D-human TM cells(3D-HTMCs) were treated daily with 500 µM H_2_O_2_or 500 µM H_2_O_2_and 0.15% iTRAB^®^(m/v) for 72 h, and molecular differences in the intracellular reactive oxygen species (iROS), state of the cells, activation of the apoptosis pathway and NF-kB and the expression ofinflammatory and fibrotic markers wereanalyzed at different time-points.Concomitant exposure significantly reduced iROS and restored TM viability, iTRAB^®^ having a significant inhibitory effect on the apoptotic pathway, activation of NF-κB, induction of pro-inflammatory (IL-1α, IL-1ß and TNFα) and pro-fibrotic (TGFβ) cytokines and the matrix metalloproteinase expressions. It is clear that this specific anti-oxidant provides a valid TM protection, suggesting iTRAB^®^ could be an adjuvant therapy in primary open-angle glaucoma (POAG).

## 1. Introduction

Polyphenols are the secondary metabolites of plants with natural antioxidants and neuro-protective properties. They belong to a family of about 5000 organic molecules present in plants, fruit, legumes, olive oil and nuts. Their application has been proven to be useful both for preventing the oxidation of biological molecules and for eliminating free radicals [1]. Indeed, recent research reported that polyphenols are involved in biochemical, cellular, and epigenetic modifications and are capable of modulating several cellular processes (e.g., redox homeostasis), as well as gene expression [2]. In particular, polyphenols contribute to mitochondrial restoration, improving the redox state homeostasis and inhibiting the trigger of the apoptosis pathway [3]. Moreover, they modulate the immune response due to their anti-inflammatory properties which down-regulate pro-inflammatory cytokines, such as IL1, TNFα and IL6 [4,5].

It has been reported that polyphenol mixtures result in greater level of protection againstoxidative damage in comparison to a single typology of anti-oxidant molecules, probably due to the synergistic action of anti-oxidant properties exerted by different molecules [6,7,8].

As is known, the drawback of an organism being aerobic is the forced reactive oxygen species (ROS) production. Indeed, even though physiological levels of ROS play a central role in redox signaling, including cell proliferation, differentiation, migration and angiogenesis, as well as the activation of enzymatic defense systems controlled either by NRF2 or NF-kB [9,10], elevated ROS formation leads to lipid, protein, nucleic acid and organelle damage. The regulation of local sub-cellular ROS concentration depends on the efficiency of generator and removal systems. Unfortunately, natural aging is characterized by the presence of senescent cells in organs, which in causing alterations in ROS homeostasis, promote tissue dysfunction. Therefore, the failure of ROS control systems results in an increased presence of pro-inflammatory cytokines, degradative proteases and growth factors which predispose to various diseases including glaucoma [11,12].

The trabecular meshwork (TM) is the most sensitive tissue of the ocular anterior chamber to oxidative stress-derived damage [13]. Therefore, it is not surprising that, once damaged, the TM is no longer able to regulate the intraocular pressure (IOP) in a conventional outflow pathway [14,15]. Thereby, the damage of TM acts as a starting point of high-tension glaucoma onset. Glaucoma is a multi-factorial neurodegenerative disease which leads to irreversible blindness. Scientific evidence supports the view that oxidative stress controls and modulates many stages of glaucoma, from its onset to its progression [3,16,17]. Either an increase in ROS production or a decrease in scavenging antioxidants promotes cellular damage which is responsible for the non-specific immune response activation.This activation increases the initiating pro-inflammatory cytokines (i.e., IL1 and TNF), triggers NF-kB signaling, thus resulting in a condition of chronic inflammation in such a way as to promote further glaucoma-related degeneration [18].

Among TM dysfunctions, the reduction of its cellularity [19], the accumulation of pro-inflammatory cytokines [20] and the loss of extracellular matrix (ECM) turnover [21], are amongst the main reasons for the alteration of its barrier function and consequently, for the increase of IOP [22,23,24]. However, the clinical trend is to lower the IOP, either by anti-hypertensive drugs or surgical practices for slowing down or stopping the glaucoma progression, real therapeutic success in the protection of the patient’s sight has not yet been reached [25,26].

Moreover, both progressive functional and gene expression alterations of TM cells lead to profound changes in the aqueous humor proteome [14]. Therefore, it is conceivable that pro-apoptotic signals, which lead to ganglion cell death, may derive from damaged TM cells. The TM-derived protein products, on reaching the posterior segment, could be crucial for the beginning of the glaucomatous cascade. In particular, these altered proteins could act both as a trigger for inner retinal layer apoptosis, of which retinal ganglion cells (RGCs) are a part, and as an activator of glia [27].

Therefore, in light of the role of oxidative stress in glaucoma, a therapeutic strategy for glaucoma treatment could be represented by the use of anti-oxidant compounds, which are able to restore or prevent the oxidative stress damage, in combination with canonical therapies. In this regard, several previous studies have shown that phyto-nutrients with a high level of biological power, e.g., polyphenols, are able to reduce the incidence of oxidative stress-associated diseases including neurodegeneration [28,29,30,31]. For a while now, polyphenols are considered asunconventional glaucoma treatment in order to suppress the neuro-inflammation process responsible for neuro-degeneration and RGC loss [3]. Therefore, the TM could represent a new promising therapeutic target in which the oxidative damage is contrasted by the local enrichment of compounds with antioxidant properties so as to provide its protection. Unfortunately, the anti-oxidant potential of dietary polyphenols, as possible sources for TM restoration, is restricted by their poor bio-availability [6]. In order to overcome this limitation, the use of the right amount of polyphenols may be given by way of eye drops.

Recently, our research group has set-up an innovative invitro model inclusive of 3D-human TM cells (HTMC) and millifluidic technology suitable for improving both cell growth and cell-to-cell contact compared to static culture models [32,33]. In this present work, we have analyzed the anti-oxidant potential of a registered trademarksolution containing high concentrations of polyphenols and fatty acids (iTRAB^®^) in counter-acting oxidative stress damage, using our 3D-advanced invitro model.

## 2. Material and Methods

### 2.1. Cell Culture

HTMC and Trabecular Meshwork Growth Medium (TMGM) were acquired from Cell APPLICATION INC. (San Diego, CA, USA). The proof of the presence of the HTMC phenotype, also after Dexamethasone treatment, was officially given by the Cell APPLICATION laboratory [34].

HTMCs were grown in TMGM and were maintained at 37 °C in a humidified atmosphere containing 5% CO_2_. However, before performing experimental treatments, in order to reduce any Fetal Bovine Serum (FBS) interference on cellular proliferation, HTMCs were cultured in low and high glucose DMEM (1:1 mix), 2mM L-glutamine, 0.5% gentamicin and 100 μg/mL streptomycin, w/o FBS—according to Keller et al. [35].

All cell cultures were found to be mycoplasma-free during regular checks with the Reagent Set Mycoplasma Euroclone (Euroclone^®^, Milan, Italy).

3D-HTMCs were obtained by suspending 5 × 10^5^ cells in 200 µL Corning^®^Matrigel^®^ Matrix (Corning Life Sciences, Tewksbury, MA, USA), and quickly seeded in bioreactor LiveBox1 (LB1) (IVTechS.r.l.,Massarosa, Italy) culture chambers [32]. After polymerization at 37 °C, 1 mL of culture media was added and then replaced with fresh medium 24 h later. The cells were then maintained under dynamic conditions for 72 h.

The dynamic culture conditions were obtained from a sophisticated model of milli-scaled multi-organ devices (IVTech, srl) in a single flow configuration (LB1, IVTechsrl) [36]. This device is described elsewhere [33].

After 24 h following seeding, the 3D-HTMCs were connected to the complete circuit (see Figure 1 in Saccà et al. 2020 [33] for the complete circuit diagram). The cell medium within the LB1 was replaced daily, while the amount of cell medium in the mixing bottle was adequately filled to avoid any nutrient depletion during the 72 h of experimental conditions. Moreover, the medium flow was maintained at the constant rate of 70 µL/mL to promote both cell survival and uniform cell distribution, as well as to overcome Matrigel^®^ degradation over time.

### 2.2. Experimental Conditions

The effects of iTRAB^®^, a concentrated mixture of polyphenols (≥2.5%) from Perilla frutescens [37] (Figure S1), on the 3D-HTMCs were investigated after prolonged oxidative stress conditions.

iTRAB^®^, which is the active principle of commercial DRAIN drops^®^,was dissolved directly into the growth medium w/o FBS and 1% DMSO (v/v) at the same concentration (0.15% m/v) as that in DRAIN drops^®^.

The 3D-HTMCs were exposed in static conditions, once a day for 2 h to 500 µM H_2_O_2_ and parallel cultures were treated with 0.15% iTRAB^®^(m/v) for another 2 h. Next, the HTMCs were cultured under dynamic conditions for 20 h to promote cell recovery [38,39]. These experimental conditions were repeatedfor 72 h.

At each end point, the 3D HTMCs were freed from the Matrigel^®^ by Corning Cell Recovery (Corning Life Sciences, NY, USA, USA), according to the manufacturer’s instructions.

### 2.3. DCF Assay

The anti-oxidant efficacy of iTRAB^®^ was evaluated on 3D HTMCs treated, as above mentioned, by dichlorofluorescein (DCF) assay in terms of ROS production.

Briefly, the 3D HTMCs were exposed to non-fluorescent 2′,7′-dichlorodihydrofluorescein diacetate (H2DCFDA, Thermo Fisher Scientific Inc., Monza, Italy, Italy), which is able to permeate the plasma membrane and is reduced to the highly fluorescent 2′,7′-dichlorofluorescein [40]. The experiments were performed as described elsewhere [32] and each condition was analyzed 6 times. DCF emission was recorded at 2hours on a fluorescent plate reader at excitation and emission wavelengths of 485 and 520 nm, respectively. The fluorescence intensity was extrapolated after subtracting the blank (Matrigel plus medium plus DCF) and the data wereexpressed as percentages of relative fluorescence units of treated vs. untreated HTMC cultures.

### 2.4. Alamar Blue Assay

During the experimental procedures, the healthy state of the 3D-HTMCs was monitored daily by AlamarBlue (AB) assay (Invitrogen^TM^, Thermo Fisher Scientific Inc., Monza, Italy) within the last 4 hour of incubation of the 20 hour of recovery time, according to the manufacturer’s instructions. Specifically, 10% AB solution (v/v) was added to the 3D-culture after 4 hours of incubation and then the resazurin reduction was quantified spectrophotometrically at wavelengths of 570 and 630 nm. The results were expressed as a number-fold of viability index changes of treated vs. untreated 3D-HTMCs.

### 2.5. Western Blotting

Cell lysates were collected using RIPA buffer (Sigma Aldrich S.r.l., Milan, Italy) adding a protease inhibitor cocktail (Complete Tablets, Roche Diagnostic GmbH, Mannheim, Germany) and sonicated until solubilized. Samples were mixed with 4x Laemmli loading buffer (Bio-Rad Laboratories, Inc., Hercules, CA, USA) and loaded onto 4–20% Tris-Glycine Gels (Bio-rad), and electrophoresis was performed for 50 min at 200 V. The protein was then transferred to a polyvinylidenedifluoride membrane (Thermo Scientific, Rockford, IL, USA) and probed overnight with the primary anti-bodies phospho-NF-kB p65, Ser 536 (Cell Signaling Technology, Danvers, MA, USA) and GAPDH (Santa Cruz Biotechnology, Santa Cruz, CA, USA), followed by incubation with HRP-conjugated secondary antibodies (NA9340V and NA931V, against rabbit and mouse primary antibodies, respectively, Amersham Life Science, Milan, Italy).The proteins were detected by Western Bright^TM^ ECL (Advansta, CA, USA), exposed to film and analyzed using a BIORAD Geldoc 2000. The presented data werecalculated after normalization with GAPDH. Densitometrical data obtained from Quantity One software (Bio-Rad) wereapplied for statistical analysis and normalized against the housekeeping GAPDH. The results were expressed as a number-fold vs. untreated cultures, respectively.

### 2.6. qPCR

Primers and probes for human IL1α, IL1β, IL6, TNFα, TGFβ, SPARC, MMP1, MMP3 and HPRT-1 were designed using the Beacon Designer 7.0 software (Premier Biosoft International, Palo Alto, CA, USA) and obtained from TibMolBiol (Genova, Italy). The sequences of PCR primers are listed in Table 1.

The total RNA was extracted using the RNeasyMicro Kit (Qiagen, Milan, Italy), according to the manufacturer’s instructions. A NanoDrop spectrophotometer (Nanodrop Technologies, Wilmington, DE, USA) was used to quantify the RNA. Then, 150 ng per sample of cDNA was synthesized by using the SuperScript^TM^ III First Strand Synthesis System (Thermo Fisher Scientific, Monza, Italy, Italy). Each PCR reaction was performed as described elsewhere [32].

Data analyses were obtained using the DNA Engine Opticon^®^ 3 Real-Time Detection System Software program (3.03 version) (Bio-Rad, Milano, Italy, Italy) and, in order to calculate the relative gene expression, compared to an untreated (control) calibrator sample, the comparative threshold Ct method [41], relative to that of the HPRT1 (the internal control), was used within the Gene Expression Analysis for iCycleriQ Real Time Detection System software (Bio-Rad, Milano, Italy, Italy) [42].

### 2.7. Apoptosis Array

The apoptosis pathway analysis was performed, as reported earlier [32], by the Human Apoptosis Antibody Array C1 (RayBio^®^; Norcross, GA, USA) in order to detect the difference between the 43-human protein expression patterns in tested cells (Table 2).

The intensity of the protein array signals was analyzed using a BIORAD Geldoc 2000 and each protein spot was normalized against Positive Control Spots printed on each membrane.

The data analysis was conducted according to the Protocol instructions of the Human Apoptosis Array C1, and the relative protein expression on different arrays was extrapolated by using the algorithm according to Human Apoptosis Array C1 protocol.

### 2.8. Statistical Analysis

Data are means ± standard deviation (SD) of the mean of at least three independent experiments. The results were analyzed using a two-way analysis of ANOVA for a single comparison or a two-way analysis of ANOVA variance followed by Bonferroni post-test for multiple comparisons. The GraphPad Prism for Windows-version 5.03 and GraphPad Software, Inc. (La Jolla, CA, USA) was used. Statistically significant differences were set at *p* < 0.05; *p* < 0.01; *p* < 0.001.

## 3. Results

### 3.1. ROS Production (Fluorimetric DCF Assay)

**Figure 1:**
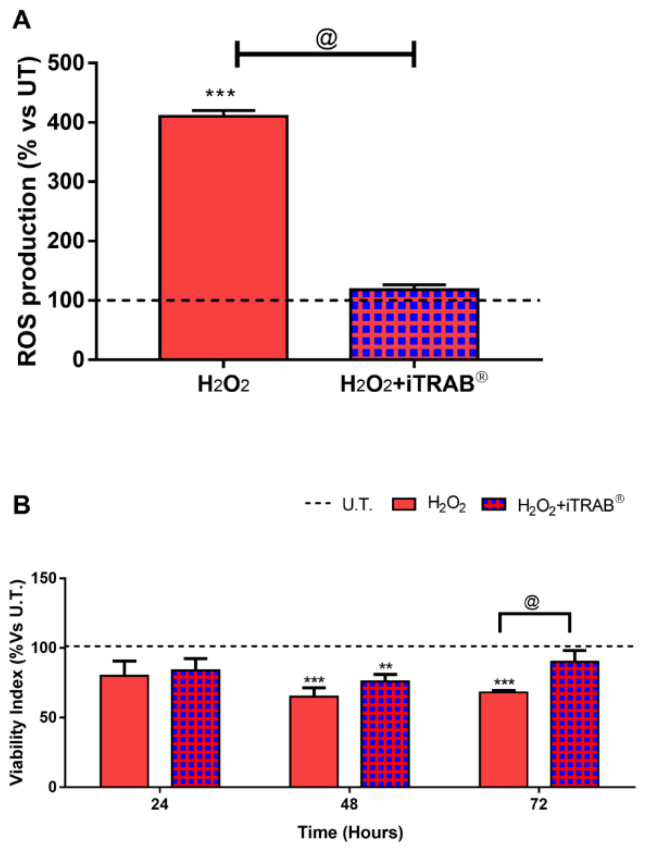
Reactive oxygen species (ROS) Production and Viability. (**A**) ROS production. Dichlorofluorescein (DCF) assays were performed after 2 h of experimental procedures. (**B**) Viability index. Viability indices were performed by Alamar blue assay. Data areexpressed as % vs. untreated (U.T) 3D-human trabecular meshwork cells (3D-HTMCs) and represent the mean ± standard deviation (SD) of 3 independent experiments. ***^,^** treated 3D HTMCs vs. UT; @ H_2_O_2_-treated vs. H_2_O_2_+iTRAB-treated 3D HTMC. ***^/@^ *p* < 0.001;** *p* < 0.01 (Two-way ANOVA followed by Bonferroni’s test).

The amount of ROS from 3D-HTMCs treated with 500 µM H_2_O_2_has only been observed to be higher by about 300% compared to either untreated 3D-HTMCs (*p* < 0.001) or those treated with 500 µM H_2_O_2_ and iTRAB^®^ (*p* < 0.001). (Figure 1A).

### 3.2. Viability Index (Alamar Blue Assay)

The viability of H_2_O_2_-treated 3D-HTMCs was significantly decreased (*p* < 0.001), starting with the 2nd exposure to the stressor. Conversely, the co-treated (H_2_O_2_ plus iTRAB^®^) 3D-HTMCs showed a time-dependent cell viability restoration at the same time points (Figure 1B).

### 3.3. Apoptosis Pathway (Human Apoptosis Antibody Array C1)

In order to corroborate the data from the viability assay, the expression of both pro-apoptotic and anti-apoptotic proteins were analyzed after the 2nd day of experimental exposures.

**Figure 2:**
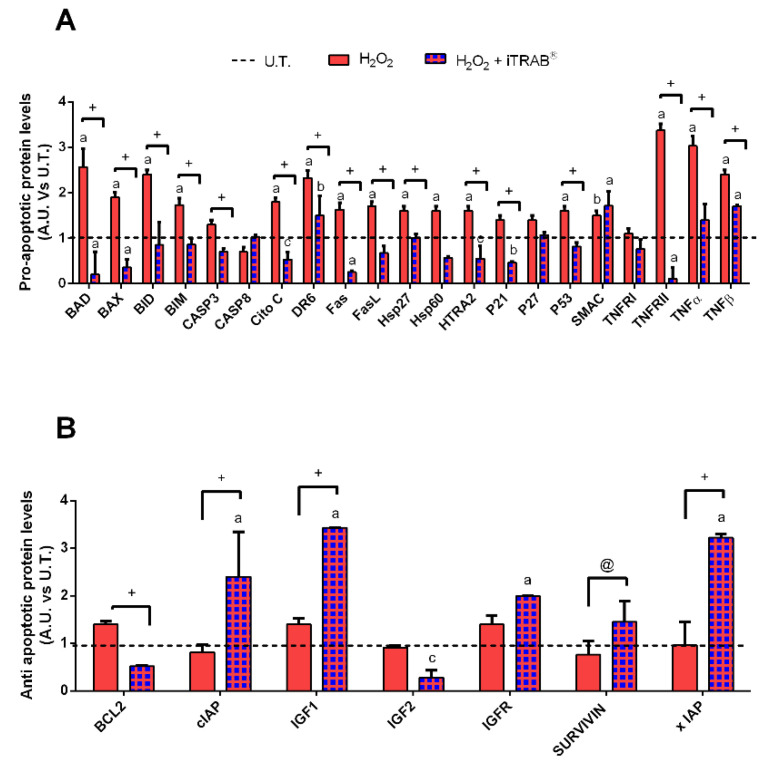
Apoptosis Array.Analysis of pro- and anti- apoptotic protein levels (**A**,**B**, respectively) in 3D-HTMCs, cultured under dynamic conditions, were performed both after the 2nd exposure of only 500 µM H_2_O_2_and co-treatment with iTRAB^®^ and 500 µM H_2_O_2_,by Human Antibody Array C1 (RayBio^®^ C-series). The black dotted line represents the protein level of untreated (U.T.) 3D human trabecular meshwork cells (HTMCs) for each protein examined. The anti-apoptotic pattern represents only the data related to those proteins that showed significant different levels compared to U.T. and H_2_O_2_-treated. Three separate conditions were arrayed and for each experiment, the intensity of Positive Control Spot was used to normalize signal responses for a comparison of results across multiple arrays. a, b, c, treated 3D HTMCs vs. UT; @, + H_2_O_2_-treated vs. H_2_O_2_+iTRAB-treated 3D HTMC. ^a/+^ *p* < 0.001; ^b^ *p* < 0.01; ^c/@^ *p* < 0.05 (Two-way ANOVA followed by Bonferroni’s test).

As shown in Figure 2A, a significant increase in the pro-apoptotic protein levels (Figure 2A) was found to a greater extent in H_2_O_2_-treated 3D HTMCs compared to the co-treated (H_2_O_2_ plus iTRAB^®^) 3D-HTMCs. However, whereas H_2_O_2_-treated 3D-HTMCs did not show changes in their anti-apoptotic protein levels (Figure 2B), in co-treated 3D-HTMCs, a significant increase in xIAP, survivin, IGFR, IGF 1 and cIAP levels wasobserved.

### 3.4. Western Blot

In our previous work, we proved that, in this in-vitro model, a stable activation of the NF-kB protein occurs after the 3rd exposure to chronic oxidative stress [32,33]. Therefore, based on such timing, we investigated the effects of iTRAB^®^ on the phospho-NF-kB p65 sub-unit modulation.

**Figure 3:**
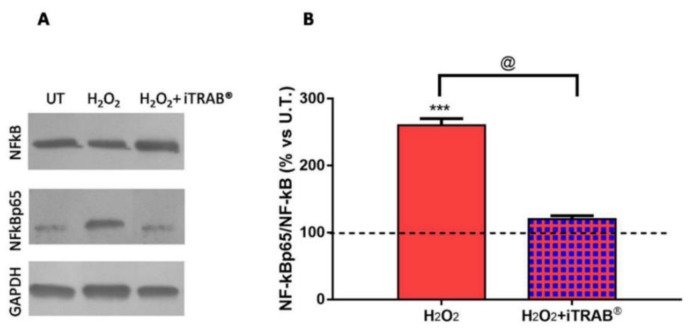
Western Blot Analysis. (**A**) The figures depicted are representative of at least three similar immunoblot analysis of NF-kB (p65), p-NF-kB (p65) protein levels in untreated 3Dhuman trabecular meshwork cells (HTMCs) and treated 3D HTMCs (H_2_O_2_/H_2_O_2_+ iTRAB^®^). GAPDH was used as an internal control for equal protein loading on the gel. (**B**) NF-kBp65 activation was evaluated in 3D-HTMCs after the 3rd exposure of 500 µM H_2_O_2_ only and co-treatment with iTRAB^®^ and 500 µM H_2_O_2_. The analysis was performed by immunoblotting and the bars represent the ratio of phosfoNF-kBp65/NF-kBp65, and are expressed as % vs. untreated HTMC cultures. Data represent the mean ± standard deviation (SD) of 3 independent experiments. The black dotted line represents the protein level of the untreated HTMC. *** treated 3D HTMCs vs. untreated (U.T.); @H_2_O_2_-treated vs. H_2_O_2_+iTRAB-treated 3D HTMC. *** *p* < 0.001; ^@^ *p* < 0.05 (Two-way ANOVA followed by Bonferroni’s test).

As reported in Figure 3, the levels of NF-kB significantly decreased (*p* < 0.05) in the 3D-HTMCs to which iTRAB^®^was added, demonstrating its anti-inflammatory effect.

### 3.5. Gene Expression Analysis

The last analysis concerned the ability of iTRAB^®^ to modulate the gene expression of 3D-HTMCs simultaneously when subjected to an oxidative stress condition. Thus, the differences in gene expression of pro-inflammatory and pro-fibrotic markers, as well as metalloproteases (MMP1 and 3), were analyzed.

**Figure 4:**
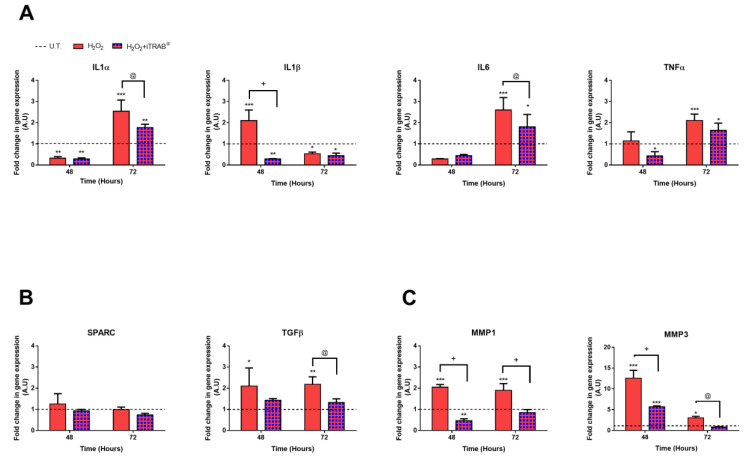
qPCR Analysis.Gene expression analysis was performed on 3D-human trabecular meshwork cells (HTMCs)HTMCs after the 2nd and 3rdexposure of 500 µM H_2_O_2_ only and co-treatment with iTRAB^®^ and 500 µM H_2_O_2_. (**A**) IL1α, IL1β, IL6, TNFα; (**B**) TGFβ and SPARC; (**C**) MMP1 and MMP3. Data are expressed as fold-increases relative to untreated cultures at the same end-point and normalized to HPRT1 housekeeping gene expression. The black dotted line represents the gene level of untreated 3D HTMCs for each gene examined. Each bar represents the mean ± standard deviation (SD) of three independent experiments performed in triplicate. ***, **, * treated 3D HTMCs vs. untreated (U.T.); +, @ H_2_O_2_-treated vs. H_2_O_2_+iTRAB-treated 3D HTMC. ***^/+^ *p* < 0.001; ** *p* < 0.01; *^/@^ *p* < 0.05 (Two-way ANOVA followed by Bonferroni’stest).

As shown in Figure 4A–C, only the 500 µM H_2_O_2_ effect triggered the inflammation pathway, according to the NF-kB analysis. Indeed, a significant up-regulation of pro-inflammatory IL1β (*p* < 0.001), pro-fibrotic TGFβ (*p* < 0.05), as well as MMP1 and MMP3 (*p* < 0.001), was already detected after the 2nd H_2_O_2_ exposure. However, after the 3rd H_2_O_2_ exposure, an increase in the other pro-inflammatory cytokines evaluated (IL1α, IL6 and TNFα) was observed, compared both to the untreated (*p* < 0.001) and co-treated cultures (*p* < 0.05). In spite of the increase in TGFβ, the SPARC expression did not show any significant modulation by the H_2_O_2_-treatment.

Conversely, the experimental conditions, in which iTRAB^®^ was administered to counteract any oxidative stress damage, showed a significant down-regulation of the above-mentioned markers, starting from the 2nd3D-HTMC exposure.

## 4. Discussion

The pathogenesis of high-tension glaucoma is characterized by oxidative stress, an elevated IOP, inflammation, an increase in ECM deposition in the outflow pathway, TM senescence, and the loss of RGCs [14,15,22,43]. In particular, a lack of an adequate anti-oxidant response, capable of counteracting the ROS production, seems to be the first issue related to typical TM dysfunction found in this form of glaucoma. Moreover, the World Health Organization (WHO) estimates that oxidative stress represents the basis for 60% of all cases of age-related blindness, including cataracts and glaucoma [44].

Currently, the only glaucoma therapeutic intervention aims to lower IOP, which does not provide a complete protection from blindness, without taking into account other possible targets [26]. In addition, the IOP-reduction with medication, except for Timolol and Dorzolamide which have antioxidant properties [45], neither restricts nor improves the oxidative stress rate [46]. Therefore, there is a need for identifying new compounds which are able to act in a therapeutic way on the several mechanisms involved in this disease.

As is known, the low anti-oxidant levels found in glaucoma patients promote an oxidative stress condition which is actually involved in glaucoma pathogenesis [22]. In fact, oxidative stress is responsible for the inflammatory condition which leads to TM damage and consequently to an outflow dysfunction [47]. In this regard, previous studies have evaluated the effect of several anti-oxidant compounds (i.e., polyphenols, carotenoids, resveratrol, vitamin E and N-acetylcysteine) in order to prevent or to counteract glaucoma [20,26,37,48,49]. In particular, the protective role of polyphenols towards the free radical attacks that affect TM has emerged, providing a greater chance of long-term visual function maintenance [1]. Indeed, these novel glaucoma approaches aim to protect the TM functionalityfrom oxidative damage for as long as possible. However, polyphenols (i.e., flavonoids), acquired from our diet, in addition to having a poor bio-availability, exert their protective effects mainly in the gastrointestinal tract [50]. Therefore, in the ophthalmology field, for instance, the topical application of polyphenolsis strongly recommended in order to increase their therapeutic efficacy.

Saccà et al. (2019) have already demonstrated the effectiveness of the anti-oxidant iTRAB^®^, that is the active principle of DRAIN drops^®^, in two-dimensional TM cells subjected to chronic oxidative stress [37].

In this present work, we have evaluated the anti-oxidant and anti-inflammatory properties of iTRAB^®^in counteracting prolonged oxidative stress with an innovative invitro model of HTMC, which combines the three-dimensional culture model (3D) with bioreactor technology [33] in order to better mimic the physiological cell behavior.

3D-HTMCs, which, in addition to 500 µM H_2_O_2_, were treated with iTRAB^®^ showed both a significant decrease of ROS production (Figure 1A) and an increase in the viability index (Figure 1B) compared to the 3D-HTMCs exposed to only 500 µM H_2_O_2_ [20,37,48]. These results highlight the anti-oxidant activity of iTRAB^®^. Moreover, the protective effects of iTRAB^®^also extend to an anti-apoptotic role: in fact, the levels of the pro-apoptotic proteins in 3D-HTMCs, exposed to both 500 µM H_2_O_2_ and iTRAB^®^, were significantly decreased, whereas the anti-apoptotic proteins were increased (Figure 2). The anti-inflammatory properties of iTRAB^®^ were investigated in 3D-HTMCs through its ability to modulate the increase in pro-inflammatory (i.e., IL-1α, IL-1β, IL-6, and TNF-α) and pro-fibrotic cytokines, as well as MMPs which are known to be associated with glaucoma [51,52].

In our 3D-Model, the exposure of TM cells to only oxidative stress led to an increase in pro-inflammatory and pro-fibrotic cytokines and MMP gene expression [51,52,53,54] (Figure 4A–C).

Although the presence of pro-inflammatory cytokines, relating to the eyes, is considered a glaucoma risk factor, in some cases (e.g., laser trabeculoplasty), pro-inflammatory cytokines can be involved in the outflow regulation, acting in synergism to increase the ECM turnover via MMPs [55]. After these preliminary considerations, it can be concluded that the chronic induction of pro-inflammatory cytokines, due to oxidative stress, contributes to glaucoma progression [56]. Indeed, TNFα up-regulation, for instance, is responsible for RGC death [57], whereas chronic IL6 activation contributes to worsening the trabecular tissue motility [58].

In our model, IL-1β mRNA expression was just significantly increased (*p* < 0.001) after the 2nd exposure to oxidative stress. However, the expressions of the other pro-inflammatory cytokines became significant only after the 3rd H_2_O_2_ exposure, maybe due to the triggering of an adaptive mechanism similar to those found in-vivo to counteract early oxidative-related damage [22,52].

This assumption was also supported by the increase in phospho-NF-kB p65 sub-unit levels (Figure 3) after the 3rd exposure to H_2_O_2_, according to our previously reported data [7,22].

In contrast, 3D-HTMCs, co-treated also with iTRAB^®^ showed significant reductions in both the phospho-NF-kB p65 sub-unit levels and the pro-inflammatory cytokine expression (*p* < 0.05). Therefore, it is reasonable to assume that iTRAB^®^anti-oxidant activity is reflected in an anti-inflammatory activity, since a decrease in the inflammatory mediators wasobserved.

Furthermore, TGFβ and metalloproteinase (MMP) gene expressions were investigated since they are involved in the characteristic TM malfunctioning, that is an impairment of the ECM metabolism and in increase in the aqueous outflow resistance [54,59,60].

As is known, the increase in the outflow resistance, found in primary open-angle glaucoma (POAG), is mainly driven by TGF-β because it is involved in both the alterations of the TM extracellular matrix homeostasis and TM cell contractility [61]. Moreover, in order to obtain further and more complete information, also the levels of SPARC, a matricellular protein which controls, and, in turn, is controlled by TGF-β, were analysed.

However, during H_2_O_2_ exposure, we observed a more marked increase of the TGF-β gene, and no significant change inSPARC expression was detected. However, the experimental conditions, which also included iTRAB^®^, was able to reduce the TGF-β levels (*p* < 0.01).

Therefore, we can speculate that, in our experimental conditions, TGF-β exerts a role as a key regulator of the cell–matrix interactions [62]. In our platform, chronic activation of IL6, due to oxidative stress, did not repress the TGF-signaling [53] but, on the contrary, its effect resulted in an increase in outflow resistance with a 3D-HTMC-contractility alteration [61].

In addition, as a result of oxidative stress-induced tissue alteration, an increase in MMP1 and MMP3 wasfound [63]. These two MMPs, which are, respectively, a collagenase [64] and a proteolytic enzyme [65], are important for ECM synthesis and its degradation.

Therefore, as a result of oxidative stress, the TM suffered, leading both to ECM alterationand its motility reduction, as observed in glaucoma.

Thus, the significant down-regulation of TGFβ and MMPs found in 3D-HTMCs, treated with both oxidative stress and iTRAB^®^, suggests that iTRAB^®^ also has a prospective role in modulating several gene expressions of genes involved in OS-induced TM damage.

In conclusion, our invitro 3D-advanced human model of TM, in providing a precise control of experimental conditions to better simulate the cell invivo micro-environment, enables the study of early oxidative stress-induced TM-alterations. Indeed, we have already demonstrated that prolonged oxidative stress-conditions induce the 3D-HTMCs to activate the inflammatory pathway rather than apoptosis [33]. Thus, in this present work, we proposed the same invitro platform as a useful tool in evaluating the anti-inflammatory and anti-oxidant effects of iTRAB^®^ as an important formulation to counteract OS-induced TM damage. In fact, the resulting data haveshown that the protection provided by the TM could represent a possible therapeutic target. Finally, iTRAB^®^, since being able to restore the TM that is the main outflow tract, could be used in combination with conventional IOP-lowering therapy.

We firmly believe that further developments of this model will lead to a better characterization of initial glaucomatous damage.

## Figures and Tables

**Table 1:** Primer sequences used for real-time quantitative polymerase chain reaction analysis.

Gene	GenBank	F	R
IL1α	NM_000575.4	CAATCTgTgTCTCTgAgTATC	TCAACCgTCTCTTCTTCA
IL1β	NM_000576.2	TgATggCTTATTACAgTggCAATg	gTAgTggTggTCggAgATTCg
IL6	NM_001318095.1	CAgATTTgAgAgTAgTgAggAAC	CgCAgAATgAgATgAgTTgTC
TNFα	NM_000594.4	GTGAGGAGGACGAACATC	GAGCCAGAAGAGGTTGAG
TGFβ2	NM_001135599.3	AACCTCTAACCATTCTCTACTACA	CgTCgTCATCATCATTATCATCA
SPARC	NM_003118.4	ATggTTCCTgTAAgCACTAA	TgAATgAATgAATgAATgAATgAC
MMP1	NM_001145938.1	ggTgATgAAgCAgCCCAgATg	CAgAggTgTgACATTACTCCAgAg
MMP3	NM_002422.5	TAATAATTCTTCACCTAAgTCTCT	AgATTCACgCTCAAgTTC
HPRT-1	NM_000194.3	GGTCAGGCAGTATAATCCAAAG	TTCATTATAGTCAAGGGCATATCC

**Table 2:** The mini map of the Human Apoptosis Array C1 (according to RayBio^®^ manufacturer’s manual).

	A	B	C	D	E	F	G	H	I	J	K	L	M	N
1	POS	POS	NEG	NEG	Blank	Blank	bad	bax	Bcl2	Bcl2-w	BID	BIM	Caspase3	Caspase8
2														
3	CD40	CD40L	cIAP2	CytoC	DR6	Fas	FasL	Blank	Hsp27	Hsp60	Hsp70	HTRA2	IGF1	IGF2
4														
5	IGFBP1	IGFBP2	IGFBP3	IGFBP4	IGFBP5	IGFBP6	IGF-1R	Livin	P21	P27	P53	SMAC	Survivn	TNF RI
6														
7	TNF RII	TNFα	TNFβ	TRAIL R1	TRAIL R2	TRAIL R3	TRAIL R4	XIAP	Blank	Blank	NEG	NEG	POS	POS
8														

## Supplementary Materials

The following are available online at https://www.mdpi.com/2077-0383/9/11/3584/s1, Figure S1: The iTRAB^®^ characterization using LC/MS as an analytical methodology.

## References

[1] Sacca S.C., Corazza P., Gandolfi S.A., Ferrari D., Sukkar S.G., Iorio E.L., Traverso C.E. (2019). Substances of Interest That Support Glaucoma Therapy. Nutrients.

[2] Leri M., Scuto M., Ontario M.L., Calabrese V., Calabrese V., Bucciantini M., Stefani M. (2020). Healthy Effects of Plant Polyphenols: Molecular Mechanisms. Int. J. Mol. Sci..

[3] Saccà S.C., Vernazza S., Iorio E.L., Tirendi S., Bassi A.M., Gandolfi S., Izzotti A. (2020). Molecular changes in glaucomatous trabecular meshwork. Correlations with retinal ganglion cell death and novel strategies for neuroprotection. Progress in Brain Research.

[4] Tabrez S., Priyadarshini M., Urooj M., Shakil S., Ashraf G.M., Khan M.S., Kamal M.A., Alam Q., Jabir N.R., Abuzenadah A.M. (2013). Cancer Chemoprevention by Polyphenols and Their Potential Application as Nanomedicine. J. Environ. Sci. Health Part C.

[5] Korhonen R., Lahti A., Kankaanranta H., Moilanen E. (2005). Nitric Oxide Production and Signaling in Inflammation. Curr. Drug Target Inflamm. Allergy.

[6] Španinger E., Hrnčič M.K., Škerget M., Knez Ž., Bren U. (2016). Polyphenols: Extraction Methods, Antioxidative Action, Bioavailability and Anticarcinogenic Effects. Molcules.

[7] De Kok T.M., Van Breda S.G., Manson M.M. (2008). Mechanisms of combined action of different chemopreventive dietary compounds. Eur. J. Nutr..

[8] Fantini M., Benvenuto M., Masuelli L., Frajese G.V., Tresoldi I., Modesti A., Bei R. (2015). In Vitro and in Vivo Antitumoral Effects of Combinations of Polyphenols, or Polyphenols and Anticancer Drugs: Perspectives on Cancer Treatment. Int. J. Mol. Sci..

[9] Imhoff B.R., Hansen J.M. (2009). Extracellular redox status regulates Nrf2 activation through mitochondrial reactive oxygen species. Biochem. J..

[10] Sies H., Jones D.P. (2020). Reactive oxygen species (ROS) as pleiotropic physiological signalling agents. Nat. Rev. Mol. Cell Biol..

[11] Gu Y., Han J., Jiang C., Zhang Y. (2020). Biomarkers, oxidative stress and autophagy in skin aging. Ageing Res. Rev..

[12] Cheng J., Liang J., Qi J. (2017). Role of nuclear factor (erythroid-derived 2)-like 2 in the age-resistant properties of the glaucoma trabecular meshwork. Exp. Ther. Med..

[13] Izzotti A., Saccà S.C., Longobardi M., Cartiglia C. (2009). Sensitivity of Ocular Anterior Chamber Tissues to Oxidative Damage and Its Relevance to the Pathogenesis of Glaucoma. Investig. Ophthalmol. Vis. Sci..

[14] Saccà S.C., Izzotti A. (2013). Focus on molecular events in the anterior chamber leading to glaucoma. Cell. Mol. Life Sci..

[15] Stamer W.D., Clark A.F. (2017). The many faces of the trabecular meshwork cell. Exp. Eye Res..

[16] Izzotti A., Bagnis A., Saccà S.C. (2006). The role of oxidative stress in glaucoma. Mutat. Res. Mutat. Res..

[17] Ahmad A., Ahsan H. (2020). Biomarkers of inflammation and oxidative stress in ophthalmic disorders. J. Immunoass. Immunochem..

[18] Vernazza S., Tirendi S., Bassi A.M., Traverso C.E., Saccà S.C. (2020). Neuroinflammation in Primary Open-Angle Glaucoma. J. Clin. Med..

[19] Alvarado J., Murphy C., Juster R. (1984). Trabecular Meshwork Cellularity in Primary Open-angle Glaucoma and Nonglaucomatous Normals. Ophthalmology.

[20] Avotri S., Eatman D., Russell-Randall K. (2019). Effects of Resveratrol on Inflammatory Biomarkers in Glaucomatous Human Trabecular Meshwork Cells. Nutrients.

[21] Acott T.S., Kelley M.J. (2008). Extracellular matrix in the trabecular meshwork. Exp. Eye Res..

[22] Saccà S.C., Izzotti A. (2008). Oxidative stress and glaucoma: Injury in the anterior segment of the eye. Progress in Brain Research.

[23] Pinazo-Durán M.D., Shoaie-Nia K., Zanon-Moreno V., Sanz-González S.M., Del Castillo F.B., García-Medina J.J. (2018). Strategies to Reduce Oxidative Stress in Glaucoma Patients. Curr. Neuropharmacol..

[24] D’Azy C.B., Pereira B., Chiambaretta F., Dutheil F. (2016). Oxidative and Anti-Oxidative Stress Markers in Chronic Glaucoma: A Systematic Review and Meta-Analysis. PLoS ONE.

[25] Overby D.R., Stamer W.D., Johnson M. (2009). The changing paradigm of outflow resistance generation: Towards synergistic models of the JCT and inner wall endothelium. Exp. Eye Res..

[26] Yang Q., Li Y., Luo L. (2018). Effect of Myricetin on Primary Open-angle Glaucoma. Transl. Neurosci..

[27] Saccà S.C., Gandolfi S., Bagnis A., Manni G., Damonte G., Traverso C.E., Izzotti A. (2016). From DNA damage to functional changes of the trabecular meshwork in aging and glaucoma. Ageing Res. Rev..

[28] Almeida S., Alves M.G., Sousa M., Oliveira P.F., Silva B.M. (2016). Are Polyphenols Strong Dietary Agents Against Neurotoxicity and Neurodegeneration?. Neurotox. Res..

[29] Mandel S., Youdim M.B. (2004). Catechin polyphenols: Neurodegeneration and neuroprotection in neurodegenerative diseases. Free. Radic. Biol. Med..

[30] D’Angelo S. (2020). Current Evidence on the Effect of Dietary Polyphenols Intake on Brain Health. Curr. Nutr. Food Sci..

[31] Bhullar K.S., Rupasinghe H.P.V. (2013). Polyphenols: Multipotent Therapeutic Agents in Neurodegenerative Diseases. Oxidative Med. Cell. Longev..

[32] Vernazza S., Tirendi S., Scarfì S., Passalacqua M., Oddone F., Traverso C.E., Rizzato I., Bassi A.M., Saccà S.C. (2019). 2D- and 3D-cultures of human trabecular meshwork cells: A preliminary assessment of an in vitro model for glaucoma study. PLoS ONE.

[33] Saccà S.C., Tirendi S., Scarfì S., Passalacqua M., Oddone F., Traverso C.E., Vernazza S., Bassi A.M. An advanced in vitro model to assess glaucoma onset. ALTEX.

[34] https://www.cellapplications.com/human-trabecular-meshwork-cells-htmc.

[35] Keller K.E., Bhattacharya S.K., Borrás T., Brunner T.M., Chansangpetch S., Clark A.F., Dismuke W.M., Du Y., Elliott M.H., Ethier C.R. (2018). Consensus recommendations for trabecular meshwork cell isolation, characterization and culture. Exp. Eye Res..

[36] Eucciferri N., Esbrana T., Ahluwalia A. (2014). Allometric Scaling and Cell Ratios in Multi-Organ in vitro Models of Human Metabolism. Front. Bioeng. Biotechnol..

[37] Sacca S.C., Pulliero A., La Maestra S., Geretto M., Profumo A., Ilderbayev O., Rosano C., Izzotti A. (2019). Protection of trabecular meshwork cells by eyedrops containing high concentration of polyphenols. New Front. Ophthalmol..

[38] Poehlmann A., Reissig K., Schönfeld P., Walluscheck D., Schinlauer A., Hartig R., Lessel W., Guenther T., Silver A., Roessner A. (2013). Repeated H2O2exposure drives cell cycle progression in aninvitromodel of ulcerative colitis. J. Cell. Mol. Med..

[39] Kaczara P., Sarna T., Burke J.M. (2010). Dynamics of H2O2 availability to ARPE-19 cultures in models of oxidative stress. Free. Radic. Biol. Med..

[40] Wang H., Joseph J.A. (1999). Quantifying cellular oxidative stress by dichlorofluorescein assay using microplate reader11Mention of a trade name, proprietary product, or specific equipment does not constitute a guarantee by the United States Department of Agriculture and does not imply its approval to the exclusion of other products that may be suitable. Free Radic. Biol. Med..

[41] Aarskog N.K., Vedeler C. (2000). Real-time quantitative polymerase chain reaction. Qual. Life Res..

[42] Vandesompele J., De Preter K., Pattyn F., Poppe B., Van Roy N., De Paepe A., Speleman F. (2009). Accurate normalization of real-time quantitative RT-PCR data by geometric averaging of multiple internal control genes. Genome Biol..

[43] Tektas O.-Y., Lütjen-Drecoll E. (2009). Structural changes of the trabecular meshwork in different kinds of glaucoma. Exp. Eye Res..

[44] Foster A., Resnikoff S. (2005). The impact of Vision 2020 on global blindness. Eye.

[45] Saccà S.C., La Maestra S., Micale R.T., Larghero P., Travaini G., Baluce B., Izzotti A. (2011). Ability of Dorzolamide Hydrochloride and Timolol Maleate to Target Mitochondria in Glaucoma Therapy. Arch. Ophthalmol..

[46] Fahmy H.M., Saad E.A.E.-M.S., Sabra N.M., El-Gohary A.A., Mohamed F.F., Gaber M.H. (2018). Treatment merits of Latanoprost/Thymoquinone – Encapsulated liposome for glaucomatus rabbits. Int. J. Pharm..

[47] Izzotti A., Longobardi M., Cartiglia C., Saccà S.C. (2011). Mitochondrial Damage in the Trabecular Meshwork Occurs Only in Primary Open-Angle Glaucoma and in Pseudoexfoliative Glaucoma. PLoS ONE.

[48] Zhao Z., Sun T., Jiang Y., Wu L., Cai X., Sun X., Sun X. (2014). Photooxidative damage in retinal pigment epithelial cells via GRP78 and the protective role of grape skin polyphenols. Food Chem. Toxicol..

[49] Bungau S., Abdel-Daim M.M., Tit D.M., Ghanem E., Sato S., Maruyama-Inoue M., Yamane S., Kadonosono K. (2019). Health Benefits of Polyphenols and Carotenoids in Age-Related Eye Diseases. Oxidative Med. Cell. Longev..

[50] Kawabata K., Yoshioka Y., Terao J. (2019). Role of Intestinal Microbiota in the Bioavailability and Physiological Functions of Dietary Polyphenols. Molecules.

[51] Almasieh M., Wilson A.M., Morquette B., Vargas J.L.C., Di Polo A. (2012). The molecular basis of retinal ganglion cell death in glaucoma. Prog. Retin. Eye Res..

[52] Luna C., Li G., Liton P.B., Qiu J., Epstein D.L., Challa P., Gonzalez P. (2009). Resveratrol prevents the expression of glaucoma markers induced by chronic oxidative stress in trabecular meshwork cells. Food Chem. Toxicol..

[53] Inoue-Mochita M., Inoue T., Kojima S., Futakuchi A., Fujimoto T., Sato-Ohira S., Tsutsumi U., Tanihara H. (2018). Interleukin-6–mediated trans-signaling inhibits transforming growth factor-β signaling in trabecular meshwork cells. J. Biol. Chem..

[54] De Groef L., Van Hove I., Dekeyster E., Stalmans I., Moons L. (2013). MMPs in the Trabecular Meshwork: Promising Targets for Future Glaucoma Therapies?. Investig. Ophthalmol. Vis. Sci..

[55] Kelley M.J., Rose A.Y., Song K., Chen Y., Bradley J.M., Rookhuizen D., Acott T.S. (2007). Synergism of TNF and IL-1 in the Induction of Matrix Metalloproteinase-3 in Trabecular Meshwork. Investig. Ophthalmol. Vis. Sci..

[56] Li G., Luna C., Liton P.B., Navarro I., Epstein D.L., Gonzalez P. (2007). Sustained stress response after oxidative stress in trabecular meshwork cells. Mol. Vis..

[57] Tezel G., Wax M.B. (2000). Increased Production of Tumor Necrosis Factor-α by Glial Cells Exposed to Simulated Ischemia or Elevated Hydrostatic Pressure Induces Apoptosis in Cocultured Retinal Ganglion Cells. J. Neurosci..

[58] Holloszy J.O., Cannon J.G. (1995). Cytokines in Aging and Muscle Homeostasis. J. Gerontol. Ser. A Boil. Sci. Med. Sci..

[59] Fleenor D.L., Shepard A.R., Hellberg P.E., Jacobson N., Pang I.-H., Clark A.F. (2006). TGFβ2-Induced Changes in Human Trabecular Meshwork: Implications for Intraocular Pressure. Investig. Ophthalmol. Vis. Sci..

[60] Vranka J.A., Kelley M.J., Acott T.S., Keller K.E. (2015). Extracellular matrix in the trabecular meshwork: Intraocular pressure regulation and dysregulation in glaucoma. Exp. Eye Res..

[61] Wang J., Harris A., Prendes M.A., Alshawa L., Gross J.C., Wentz S.M., Rao A.B., Kim N.J., Synder A., Siesky B. (2017). Targeting Transforming Growth Factor-β Signaling in Primary Open-Angle Glaucoma. J. Glaucoma.

[62] Frangogiannis N.G. (2012). Matricellular Proteins in Cardiac Adaptation and Disease. Physiol. Rev..

[63] Nagase H. (1996). Matrix metalloproteinases. Zinc Metalloproteases in Health and Disease.

[64] Rohani M.G., Parks W.C. (2015). Matrix remodeling by MMPs during wound repair. Matrix Biol..

[65] Stetler-Stevenson W.G. (2001). The Role of Matrix Metalloproteinases in Tumor Invasion, Metastasis, and Angiogenesis. Surg. Oncol. Clin. N. Am..

