# Supplementary figures and images for: Can Polyphenols in Eye Drops Be Useful for Trabecular Protection from Oxidative Damage?
Source: J Clin Med. 2020 Nov 6;9(11):3584. doi: 10.3390/jcm9113584 (PMC7694784; doi:10.3390/jcm9113584)

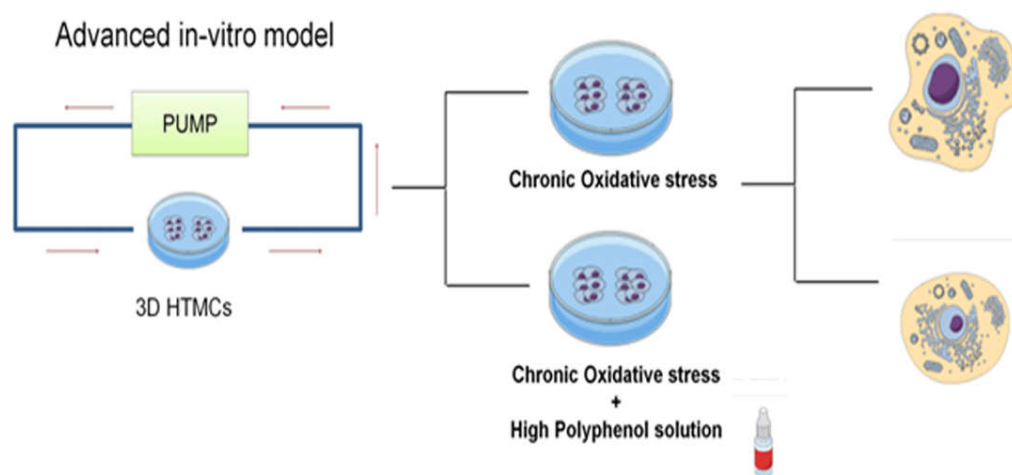

**Figure 1.** The iTRAB® characterization using LC/MS as an analytical methodology.

Supplement: Supplementary file 1 [file jcm-09-03584-s001.pdf]
